# Si Shen Wan Inhibits mRNA Expression of Apoptosis-Related Molecules in p38 MAPK Signal Pathway in Mice with Colitis

**DOI:** 10.1155/2013/432097

**Published:** 2013-10-08

**Authors:** Hai-Mei Zhao, Xiao-Ying Huang, Feng Zhou, Wen-Ting Tong, Pan-Ting Wan, Min-Fang Huang, Qing Ye, Duan-Yong Liu

**Affiliations:** ^1^Jiangxi University of Traditional Chinese Medicine, Nanchang, Jiangxi 330004, China; ^2^Jiangxi University of Traditional Chinese Medicine, Science and Technology College, 666 Fusheng Road, Nanchang, Jiangxi 330025, China; ^3^Jiangxi University of Traditional Chinese Medicine, School of Computer, 18 Yunwan Road, Nanchang, Jiangxi 330004, China

## Abstract

Si Shen Wan (SSW) is used to effectively treat ulcerative colitis (UC) as a formula of traditional Chinese medicine. To explore the mechanism of SSW-inhibited apoptosis of colonic epithelial cell, the study observed mRNA expression of apoptosis-related molecules in p38 MAPK signal pathway in colonic mucosa in colitis mice treated with SSW. Experimental colitis was induced by 2,4,6-trinitrobenzene sulfonic acid (TNBS) in mice; meanwhile, the mice were administrated daily either SSW (5 g/kg) or p38 MAPK inhibitor (2 mg/kg) or vehicle (physiological saline) for 10 days. While microscopical evaluation was observed, apoptosis rate of colonic epithelial cell and mRNA expression of apoptosis-related molecules were tested. Compared with colitis mice without treatment, SSW alleviated colonic mucosal injuries and decreased apoptosis rate of colonic epithelial cell, while the mRNA expressions of p38 MAPK, p53, caspase-3, c-jun, c-fos, Bax, and TNF-**α** were decreased in the colonic mucosa in colitis mice treated with SSW, and Bcl-2 mRNA and the ratio of Bcl-2/Bax were increased. The present study demonstrated that SSW inhibited mRNA expression of apoptosis-related molecules in p38 MAPK signal pathway to downregulate colonic epithelial cells apoptosis in colonic mucosa in mice with colitis.

## 1. Introduction

Ulcerative colitis (UC) is a chronic, inflammatory disease of colonic mucosa characterized by a relapsing-remitting course. Although the exact pathogenesis of UC is unclear, it is well known that excessive apoptosis with insufficient proliferation in crypt proliferative zones has been proposed as a mechanism for mucosal ulceration in UC [1]. Increased apoptosis of colonic epithelial cells in the acute inflammatory sites was a hallmark of ulcerative colitis [2]. Apoptosis of colonic epithelial cells was induced by using murine models with DSS- or TNBS-induced colitis in previous studies [3, 4]. Apoptosis of colonic epithelial cells can disrupt intestinal mucosal integrity and barrier function and lead to other changes associated with colitis. Therefore, inhibiting apoptosis of colonic epithelial cells will be one of the main attempts to treat UC [5].

Mitogen-activated protein kinases (MAPKs), a family of serine/threonine kinases, encompass the extracellular signal-regulated kinases, p38 MAPKs, and so on [6]. Many studies had provided evidence that p38 MAPK activation was responsible for apoptosis of colonic epithelial cells in ulcerative colitis [7]. The primary pathways to control apoptosis were correlative with the function of p38 MAPK (i.e., reinforcing expression of c-myc [8], phosphorylating p53 [9], participating Fas/Fas L signal [10], activating c-jun and c-fos [11], inducing transposition of Bax [12], augmenting production of TNF-*α* [13], and ect.)

Si Shen Wan (SSW) is a famous traditional Chinese herbal medicine formula, which was used to treat UC, allergic colitis, chronic colitis, and so on [14, 15]. The reported effective rate of SSW was above 90% when it was used to treat chronic colitis by oral administration or enema [16]. But the mechanism of SSW is unclear. In our previous studies, we had demonstrated that SSW effectively alleviated colonic injury of rats with experimental colitis, regulated colonic epithelial cell cycle, and inhibited expression of Fas in colonic mucosa [17, 18]. However, the pathway is illdefined that SSW inhibited apoptosis to protect colonic epithelial cells in treatment of UC.

## 2. Materials and Methods

### 2.1. Animals

C57/BL mice (half males and half females) weighting 22–26 g (the animal certificate number was SCXK 2009-0004) were purchased from Sino-British Sippr/BK Laboratory Animal Co. Ltd. (Changsha, China). The animals were caged at 20 ± 1°C with a humidity of 50% ± 5% in a 12 h light/dark cycle. Standard diet and water were provided ad libitum throughout the experiments. The animals were acclimatized for 3 days before experiments and handled according to the Guidelines of the Jiang Xi University of Traditional Chinese Medicine Animal Research Committee. These mice were randomly assigned to 4 groups: the Normal group (mice were induced and administrated by physiological saline), the TNBS 10 d group (mice were induced by TNBS and administrated by physiological saline), the TNBS 10 d + SSW group (mice were induced by TNBS and treated with SSW), and the TNBS 10 d + SB203580 group (mice were induced by TNBS and treated with p38 MAPK inhibitor (SB203580)). Ten animals were in each group.

### 2.2. Drugs

SSW is a traditional Chinese herbal medicine formula, which is composed of *Evodia rutaecarpa* (Juss.) Benth (Wu Zhu Yu), *Psoraleacorylifolia* L. (Bu Gu Zhi), Fructus *Schisandra chinensis* (Turcz.) Baill. (Wu Wei Zi), *Myristica fragrans* Houtt. (Rou Dou Kou), *Zingiber officinale* Rosc. (Sheng Jiang), and *Ziziphus Jujuba* Mill. (Da Zao). All medicinal herbs were purchased from Huang Qing Ren Drugstore (Nanchang, China) and identified by professor Xiao-lan Chu of Jiangxi University of Traditional Chinese Medicine. The major identified effective phytochemical compound of each herb included in SSW is illustrated in Table 1. 2,4,6-trinitrobenzene sulfonic acid (TNBS) (batch number 2508-19-2) and p38 MAPK inhibitor (SB203580, batch number 152121-47-6) were from Sigma-Aldrich, St. Louis, MO, USA.

### 2.3. Trinitrobenzene Sulfonic Acid-Induced Colitis

According to the previous study [19], the experimental colitis was induced by TNBS in mice. Briefly, after 12 h absolute diet, the mice were lightly anesthetized with pentobarbital and administrated TNBS solution (100 mg·kg^−1^ body TNBS was dissolved in 0.15 mL of 30% ethanol) by enema. The freshly prepared solution was injected into the colon 4 cm proximal to the anus by a plastic hose tube (the diameter is 1.5 mm). The mice were maintained in a head-down position for 1 min.

### 2.4. Preparation of SSW Powder and Therapeutic Protocol

All medicinal herbs were extracted twice by refluxing with water (1 : 10, v/v) for 1 h per time. The water extract was filtered and evaporated by rotary evaporation below 60°C under reduced pressure. And then the residue was freeze-dried; the powder was taken (25.2% w/w of the crude drugs), stored at 4°C, and dissolved at the desired concentration with distilled water before use [20]. The quality control of SSW extract was reported in our previous article [20]. And SSW powder in the present study was the same batch as in our previous study. 

The animals in the TNBS 10 d + SSW group received SSW extracts daily by gavage at a dose of 5 g/kg for 10 days, while the mice in the TNBS 10 d + SB203580 group were injected intraperitoneally with 2 mg/kg p38 MAPK inhibitor. And the animals in the Normal and TNBS 10 d group received the equivalent volume of physiological saline.

### 2.5. Microscopical Evaluation

On day 11, all animals were killed after anesthesia with intraperitoneally administrated urethane (2.0 g/kg). The colon of mouse was separated rapidly into two parts. One part was used to test apoptosis rate of colonic epithelial cell and to isolate total RNA. The other specimens were processed for paraffin sectioning and hematoxylin-eosin (HE) staining (*n* = 8). Histological injuries were evaluated according to the previously described scales [20, 21], taking into consideration both inflammatory cell infiltration and tissue damage. Scores for infiltration: 0, no infiltration; 1, increased number of inflammatory cells in the lamina propria; 2, inflammatory cells extending into the submucosa; 3, transmural inflammatory infiltrates; and for tissue damage: 0, no mucosal damage; 1, discrete epithelial lesions; 2, erosions or focal ulcerations; 3, severe mucosal damage with extensive ulceration extending into the bowel wall.

### 2.6. Apoptosis Rate of Colonic Epithelial Cell Was Analyzed by Flow Cytometry (FCM)

The colonic tissue (*n* = 8) was clipped into fragments in icebath and filtrated to collect colonic epithelial cells. These cells were washed in cold phosphate-buffered saline (PBS), recentrifuged, and resuspended in annexin-binding buffer. The cell density was determined and diluted into 1 × 10^6^ cells/mL. 5 *μ*L Alexa Fluor 488 annexin V and 1 *μ*L 100 *μ*g/mL PI (propidium iodide) working solution was added to each 100 *μ*L of cell suspension. After the incubation for 15 minutes at room temperature, the stained cells were analyzed by flow cytometry at 530 nm.

### 2.7. Real-Time-Polymerase Chain Reaction (PCR)

Assessment of mRNA was performed by real-time-polymerase chain reaction according to the previous study [22]. Total RNA (*n* = 5) was isolated from fresh full-thickness colonic tissue by using Trizol reagent (Invitrogen Life Technologies Co. Ltd., USA) method as described earlier. Total RNA aliquots were reverse transcribed to assay for p38 MAPK, p53, c-myc, c-jun, c-fos, caspase-3, Bax, Bcl-2, and TNF-*α*. The thermal cycle involved a 3-minute hot start at 95°C, followed by 40 cycles at 95°C (15 seconds), 60°C (20 seconds), 72°C (20 seconds), and 82°C (20 seconds). Primers, annealing temperatures, and products length used for each gene are shown in Table 2. All resulting products were analyzed by agarose gel electrophoresis (visual absence of significant 28S and 18S band 3degradation) and quantified by spectrophotometry on a Bio-Rad Gel Doc 1000 (BioRad, Hercules, CA, USA). Results were standardized to GAPDH.

### 2.8. Statistical Analysis

All parameters were expressed as mean ± standard deviation (SD). For comparison of >2 conditions, a one-way analysis of variance (ANOVA) with Tukey post hoc test was used by SPSS 13.0 software (SPSS Inc., Chicago, IL, USA). Differences with *P* < 0.05 were considered significant.

## 3. Results

### 3.1. SSW Alleviates the Colonic Mucosal Injuries Induced by TNBS

**Figure 1:**
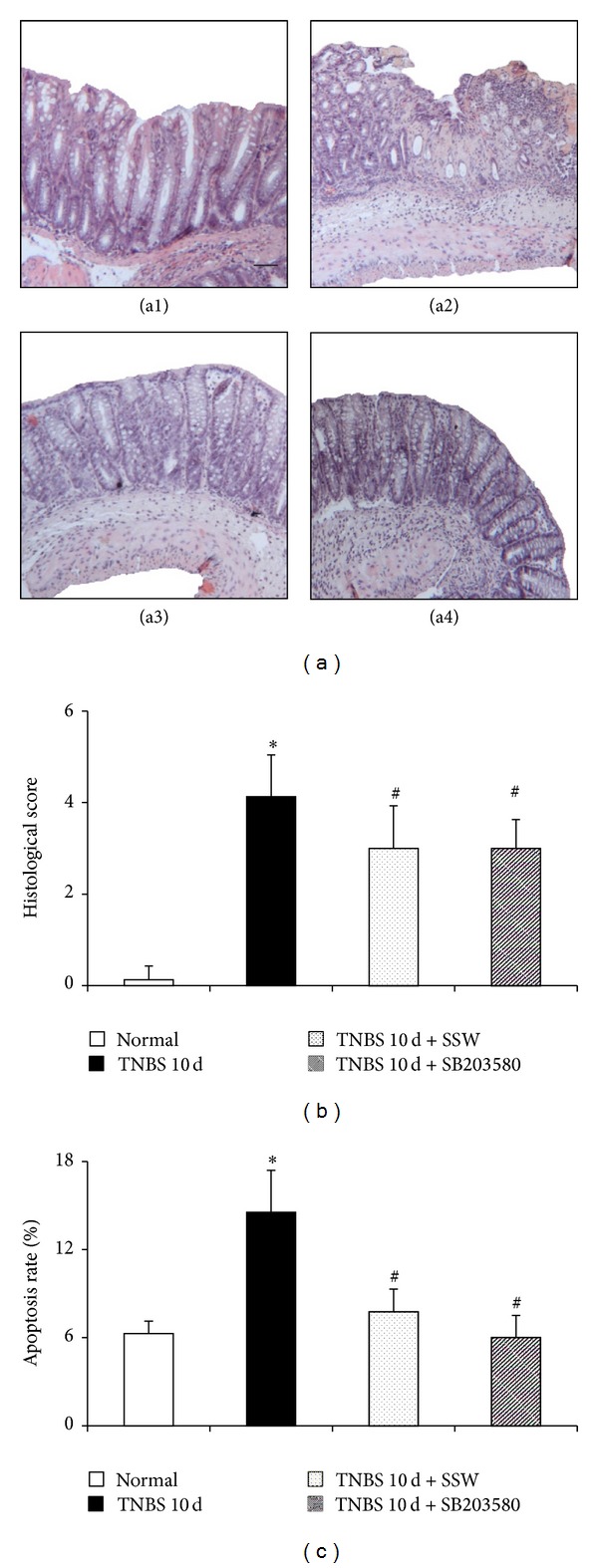
Representative histological images and scores and apoptosis rate of colonic epithelial cell. (a) Representative histological images stained by HE: (a1) Normal (mice were induced and administrated by physiological saline), (a2) TNBS 10 d (mice were induced by TNBS and administrated by physiological saline), (a3) TNBS 10 d + SSW (mice were induced by TNBS and treated by SSW), and (a4) TNBS 10 d + SB203580 (mice were induced by TNBS and treated with p38 MAPK inhibitor (SB203580)); Bar = 100 *μ*m. (b) Histological scores. (c) Apoptosis rates. Data were mean ± SD (*n* = 8). *P < 0.05 versus Normal group; ^#^ *P* < 0.05 versus TNBS 10 d group.

These colonic mucosal injuries persisted till day 10, including epithelial necrosis, epithalaxy, impaired mucosa involving submucosa with hyperemia and edema, and ulcer accompanied with numerous inflammatory cell infiltrations (Figure 1(a), (a2)), but alleviated by treatment with SSW (Figure 1(a), (a3)). The colonic histological injury scores were higher (Figure 1(b)) in TNBS 10 d groups compared to Normal groups, all of which were ameliorated significantly by SSW treatment. These results revealed a significant improvement of histology in TNBS 10 d + SSW group compared with TNBS alone group.

### 3.2. SSW Decreases Apoptosis Rate of Colonic Epithelial Cell in Mice with Colitis

Excessive apoptosis of colonic epithelial cell is an important event in the pathogenesis of ulcerative colitis. In the present study, compared with the Normal group, apoptosis rate of colonic epithelial cell in mice in the TNBS 10 d group was elevated significantly (Figure 1(c)) (*P* < 0.05). This enhancement was blunted significantly by SSW and SB203580 treatment (Figure 1(c)) (*P* < 0.05).

### 3.3. SSW Inhibits mRNA Expression of Apoptosis-Related Molecules in p38 MAPK Signal Pathway in Mice with Colitis

**Figure 2:**
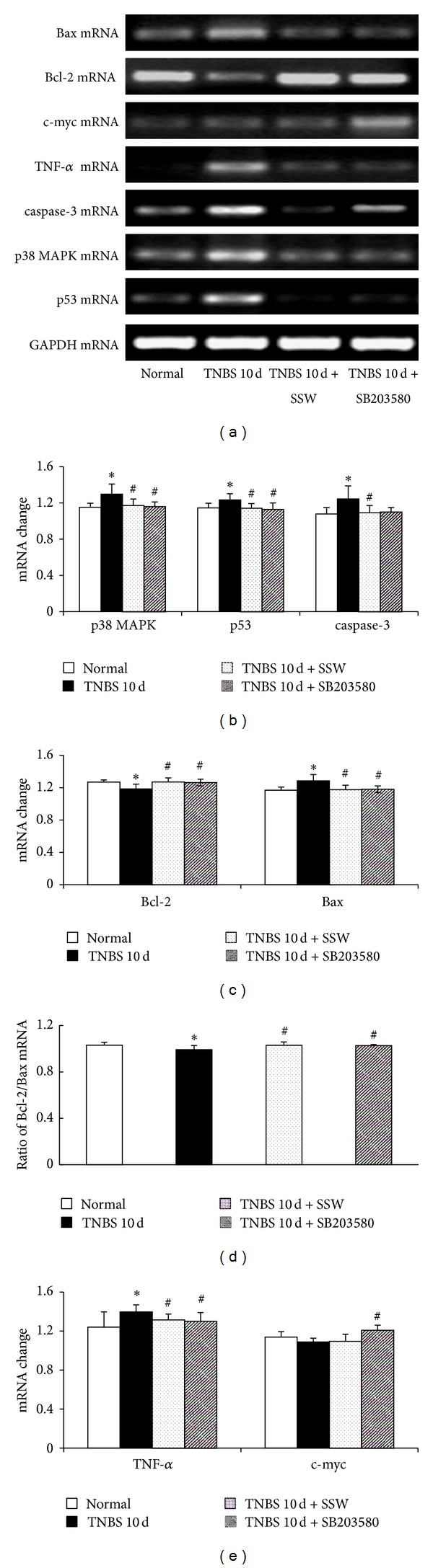
mRNA expression of p38 MAPK, p53, caspase-3, TNF-*α*, c-myc, Bcl-2, and Bax. (a) Representative electrophoretograms of p38 MAPK, p53, caspase-3, TNF-*α*, c-myc, Bcl-2, Bax, and GAPDH; (b) p38 MAPK, p53, and caspase-3 mRNA change; (c) Bcl-2 and Bax mRNA change; (d) ratio of Bcl-2/Bax mRNA change; (e) TNF-*α* and c-myc mRNA change. Data were mean ± SD (*n* = 5). *P < 0.05 versus Normal group; ^#^ *P* < 0.05 versus TNBS 10 d group.

**Figure 3:**
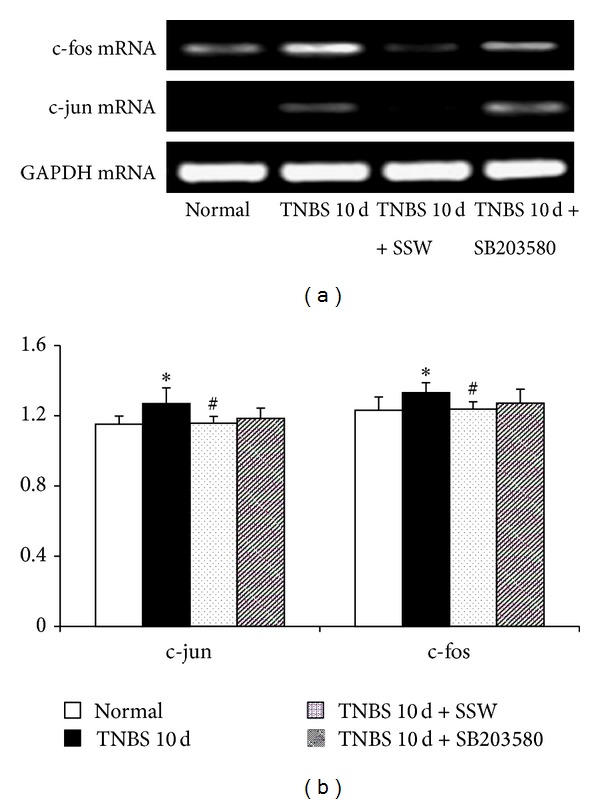
mRNA expression of c-jun and c-fos. (a) Representative electrophoretograms of c-jun, c-fos, and GAPDH. (b) c-jun and c-fos mRNA change. Data were mean ± SD (*n* = 5). *P < 0.05 versus Normal group; ^#^ *P* < 0.05 versus TNBS 10 d group.

To investigate the mechanism of SSW in regulating apoptosis of colonic epithelium to protect colonic mucosa, the study principally observed mRNA expression of apoptosis-related molecules in p38 MAPK signal in colonic mucosa. As seen in Figures 2 and 3, compared with the Normal group, the expression of p38 MAPK (Figures 2(a) and 2(b)), p53 (Figures 2(a) and 2(b)), TNF-*α* (Figures 2(a) and 2(e)), caspase-3 (Figures 2(a) and 2(b)), c-jun (Figures 3(a) and 3(b)), c-fos (Figures 3(a) and 3(b)), and Bax mRNA (Figures 2(a) and 2(c)) was remarkably increased in the colonic mucosa in mice in the TNBS 10 d group (*P* < 0.05), while Bcl-2 mRNA expression (Figures 2(a) and 2(c)) and the ratio of Bcl-2/Bax (Figures 2(a) and 2(d)) were transparently decreased (*P* < 0.05). However, the expression of p38 MAPK (Figures 2(a) and 2(b)), p53 (Figures 2(a) and 2(b)), caspase-3 (Figures 2(a) and 2(b)), c-jun (Figures 3(a) and 3(b)), c-fos (Figures 3(a) and 3(b)), Bax (Figures 2(a) and 2(c)), and TNF-*α* mRNA (Figures 2(a) and 2(c)) in the TNBS 10 d + SSW group was lower than in the TNBS 10 d group (*P* < 0.05), but Bcl-2 mRNA expression (Figures 2(a) and 2(c)) and the ratio of Bcl-2/Bax (Figures 2(a) and 2(d)) were upregulated (*P* < 0.05). All results had shown that overexpression of mainly apoptosis genes in p38 MAPK signal pathway existed in the pathogenesis of UC and was inhibited after treatment by SSW. 

## 4. Discussion

The evidences of SSW therapeutic effects were reported in our previous study including decreased colon wet weight, colon organ coefficient, and colonic damage score; shorted colon length; improved pathological injury [20]. In the present study, TNBS administration successfully induced colonic mucosal injuries in mice, as evidenced by microscopic manifestations. Significantly, apoptosis rate of colonic epithelial cells was increased in the TNBS 10 d group and was inhibited by SSW treatment. It demonstrated that its therapeutic effects on TNBS-induced colonic mucosal injury were intimately related to inhibited apoptosis of colonic epithelial cells.

Increased apoptosis of colonic epithelial cells in crypt enterocytes plays an important role in the pathogenesis of UC [19]. The p38 MAPK signal pathway was closely related to apoptosis of colonic epithelial cells in UC colonic mucosa [7]. In the present study, p38 MAPK mRNA was overexpressed in colitis mice induced by TNBS, while the expression of p53, caspase-3, c-jun, c-fos, Bax, and TNF-*α* mRNA was increased in the colonic mucosa of mice from the model group. These molecules are wellknown and are important signals to induce/inhibit apoptosis in p38 MAPK signal pathway. On one hand, these facts have shown that the p38 MAPK signal pathway was activated and played a crucial role in the pathogenesis of colitis induced by TNBS, and on the other hand, the results were coincident with many documents [7, 23]. 

Others researchers had indicated their correlations with p38 MAPK and abovementioned factors. (1) p38-MAPK is necessary for Bax activation and apoptosis in vitro [12]. Bax seems to be necessary for induction of apoptosis, and Bcl-2 is a suppressor gene of apoptosis. Increased expression of Bcl-2 can combine with Bax to form more stable heterodimers to inhibit apoptosis, so the ratio of Bcl-2/Bax can regulate apoptosis [24]. (2) Expression of TNF-*α* and TNF-*α* receptor-mediated signaling is required for p38 activation [16]. (3) The p38 MAPK signaling cascades in the regulation of AKT-dependent cyclin D1 and c-myc internal ribosome entry site (IRES) activity, while as a downstream of p38 MAPK signaling, IRES mediated the synthesis of c-myc during apoptosis [8]. (4) As an important marker of apoptosis, caspases, Bcl-2, and p53 proteinase family related genes were activated after Fas and Fas L were integrated in the initial phase, while overexpression of caspases subsequently activated MAPKs signal pathway to phosphorylate p38 and c-jun and expressed transcription factor (p53 and Fas L) to induce apoptosis [25, 26]. 

In the present study, SSW had inhibited the expression of p38 MAPK mRNA in colonic mucosa in colitis mice. The results had hinted that activation of p38 MAPK signal was restrained by SSW. Furthermore, SSW had refrained expression of p53, caspase-3, c-jun, c-fos, Bax, and TNF-*α* mRNA and improved the level of Bcl-2 mRNA and the ratio of Bcl-2/Bax in colitis mice treated with SSW. Our previous studies had proved that SSW inhibited the expression of Fas to decrease apoptosis of colonic epithelial cells in colitis mice treated with SSW [17, 18]. And in our previous study, SSW increased the expression of IL-4 and IL-10 mRNA which inhibit the production of proinflammatory factor (TNF-*α*, IL-1) as anti-inflammatory cytokine [20]. In addition, the c-myc can keep dynamic equilibrium between cell proliferation and apoptosis [27]. The activated c-myc improves growth vigor of cell to restore injury and induce cell apoptosis [27]. The phenomenon is coincident with the pathogenesis of UC which is excessive in inflammatory cell proliferation and epithelial cell apoptosis [1, 2]. While p38 MAPK inhibitor (SB203580) increased expression of c-myc mRNA in some situation as activated PTEN [8]. However, it is not an emphasis in the present study, but is worth studying that the recovery effect of p38 MAPK inhibitor is or not related with over-expression of c-myc mRNA. In conclusion, SSW effectively inhibited mRNA expression of apoptosis-related molecules in p38 MAPK signal pathway to downregulate colonic epithelial cells apoptosis in colonic mucosa in mice with colitis.

## Figures and Tables

**Table 1:** Characterization of the herbs included in Si Shen Wan.

Herbs	Percentage content (%)	Identified compounds	Effects	References
*Evodia rutaecarpa* (Juss.) Benth (Wu Zhu Yu)	6.67	Evodiamine rutaecarpine	Bacteriostasis, analgesia, antiemetic	[28]
*Psoralea corylifolia* L. (Bu Gu Zhi)	26.67	Psoralen isopsoralen	Anti-aging, antineoplastic	[29]
Fructus *Schisandra chinensis* (Turcz.) Baill. (Wu Wei Zi)	13.33	Schisandrin	Anti-free radical, boost immunity	[30]
*Myristica fragrans* Houtt. (Rou Dou Kou)	13.33	Ursolic acid	Bacteriostasis, antineoplastic	[31]
*Zingiber officinale* Rosc. (Sheng Jiang)	26.67	Gingerol	Gastroprotective effects	[32]
*Ziziphus Jujuba* Mill. (Da Zao)	13.33	Polysaccharides	Antioxidative, antiglycative, antiapoptotic efects	[33]

**Table 2:** Primer sequences for RT-PCR (mouse).

Gene	Primer sequences	Annealing temperature (°C)	Products (bp)
GAPDH	F: 5′GGAAAGCTGTGGCGTGAT3′	59	308
	R: 5′AAGGTGGAAGAATGGGAGTT3′		
caspase-3	F: 5′GCTGGACTGCGGTATTGAGA3′	59	142
	R: 5′CCATGACCCGTCCCTTGA3′		
p38MAPK	F: 5′GACGAATGGAAGAGCCTGAC3′	59	260
	R: 5′AGATACATGGACAAACGGACA3′		
TNF-*α*	F: 5′CTCAGCCTCTTCTCATTCCT3′	59	101
	R: 5′ATTTGGGAACTTCTCCTCCT3′		
Bcl-2	F: 5′TGGGATGCCTTTGTGGAAC3′	59	167
	R: 5′CATATTTGTTTGGGGCAGGTC3′		
Bax	F: 5′TGCTACAGGGTTTCATCCAG3′	59	175
	R: 5′ATCCACATCAGCAATCATCC3′		
c-fos	F: 5′TGCGTTGCAGACCGAGA3′	59	293
	R: 5′GAAACAAGAAGTCATCAAAGGG3′		
p53	F: 5′TGCTGAGTATCTGGACGACA3′	59	166
	R: 5′CAGCGTGATGATGGTAAGG3′		
c-jun	F: 5′GGCTGTTCATCTGTTTGTCTTCA3′	59	300
	R: 5′TTCTTTACGGTCTCGGTGGC3′		
c-myc	F: 5′GCTCAAAGCCTAACCTCACAA3′	59	117
	R: 5′AAAGAAAGAAGATGGGAAGCA3′		
